# Assessment of the Clinical Diagnosis of Onychomycosis by Dermoscopy

**DOI:** 10.3389/fsurg.2022.854632

**Published:** 2022-03-16

**Authors:** Yan Ma, Ying Ji, Wen Cen, Zusha Qiao, Yan Gao, Lu He, Wenli Feng

**Affiliations:** ^1^Department of Dermatology, The Second Hospital of Shanxi Medical University, Taiyuan, China; ^2^Key Research Lab of Airway Neuroimmunology, Taiyuan, China; ^3^Department Social Medicine, School of Public Health, Shanxi Medical University, Taiyuan, China

**Keywords:** onychomycosis, dermoscopy, fluorescence microscopy, sensitivity, diagnosis

## Abstract

**Background:**

As a common clinical superficial fungal infection, the diagnosis of onychomycosis relies on clinical features, traditional KOH direct microscopy and fungal culture. In recent years, dermoscopy has been widely used in the diagnosis and treatment of infectious diseases and has provided new options for the diagnosis of onychomycosis.

**Objective:**

To evaluate the value of dermoscopy in the clinical diagnosis of onychomycosis and to explore the relationship between each clinical subtype and the dermoscopic pattern.

**Methods:**

A retrospective study of 114 cases of clinically suspected onychomycosis was conducted to compare the differences between dermoscopy and fungal pathogenic examination (microscopy and culture) in the diagnostic sensitivity of onychomycosis and to analyze the relationship between nine common dermoscopic modalities and clinical subtypes of onychomycosis.

**Results:**

Among the 114 proposed patients, 87 nails with positive fluorescent staining microscopy and/or positive fungal cultures were diagnosed as onychomycosis. The sensitivity and specificity of dermatoscopy, using the mycological findings as a reference, were 86.21 and 33.33%, respectively. The incidence of common dermatoscopic patterns in the 87 nails with confirmed onychomycosis was as follows: white flocculation in 76 cases (87.35%), longitudinal nail pattern in 72 cases (82.76%), jagged changes in the distal nail plate in 69 cases (79.31%) and yellow staining in 46 cases (52.87%), these four patterns were more commonly seen in the distal lateral subungual onychomycosis and total dystrophic onychomycosis, but there was no statistical difference in the positive dermatoscopic pattern between these two types (*P* > 0.05).

**Conclusion:**

Dermoscopy can be an important aid in the diagnosis of onychomycosis, especially when fungal microscopy or culture is not appropriate, but this method is still not a substitute for fungal microscopy and culture.

## Introduction

Onychomycosis is a fungal infection of the fingernails caused by dermatophytes, non-dermatophytes and yeasts, with a global prevalence ranging from 2 to 13% in all age groups (1). Onychomycosis accounts for ~50% of all nail diseases (2) and 30% of superficial fungal diseases (3), it is also the most common nail disease in the clinic, nail trauma, poor life hygiene habits, and concomitant autoimmune diseases can easily lead to the development of nail fungus, in addition to its occurrence is also associated with factors such as economic and work stress, social exclusion and reduced quality of life (4, 5). Onychomycosis is mainly characterized by changes in the shape and color of the nail, affecting the patient's image and even secondary social difficulties, and because it is difficult to eradicate it can easily become a source of recurrent infections of other superficial fungal diseases. The rate of positive tests for nail fungal pathogenesis is the lowest of all superficial fungal diseases and is the most difficult type to diagnose and takes longer to administer, while accurate diagnosis is essential for the ongoing treatment and management of nail fungal disease (5, 6). The most widely used diagnostic method for nail fungal disease is still the traditional 10% KOH (potassium hydroxide) direct microscopy and fungal culture, however, its diagnostic sensitivity and specificity depends on the level of the examiner and the examination equipment, and the detection rate is <50% (7). Therefore, the search for other means to diagnose or aid in the diagnosis of the disease is a current clinical concern.

With the development of science and technology, new tests and examinations are emerging (6, 8–10). When comparing the advantages and disadvantages of PAS staining of nail tissue, KOH, and fungal culture in the diagnosis of onychomycosis, Haghani (11) used positive fluorescent staining as the diagnostic criterion for nail fungal infection. Modern dermoscopy, as a non-invasive, easy-to-use, and timely return portable examination tool, is a new and important adjunct to clinical work in dermatology and diagnostic methods, and has been widely used in China in recent years, and more and more physicians are using dermoscopy for onychomycosis in patients who are not suitable for routine fungal microscopy or culture (12, 13). In this study, we observed the use of dermoscopy in the diagnosis of onychomycosis in Shanxi, China.

## Materials and Methods

We used a retrospective study of patients with a proposed diagnosis of onychomycosis in our outpatient clinic from September 2017 to May 2018. Those who received antifungal medication in the last 6 months were excluded, and the largest lesion area was selected when multiple toenails were involved in the same patient. Each participant was given a detailed medical history, and gender, age, site of onset, number of nails, morphological characteristics and clinical subtypes were recorded separately, and the information recorded was classified and organized. All diseased nails were perfected with clinical staging observations, calcofluor white (CFW) fungal microscopy, fungal culture and dermoscopy. The study was conducted in accordance with the principles of the Declaration of Helsinki and was approved by the local medical ethics committee. All patients were informed about the study and signed an informed consent form.

**Figure 1:**
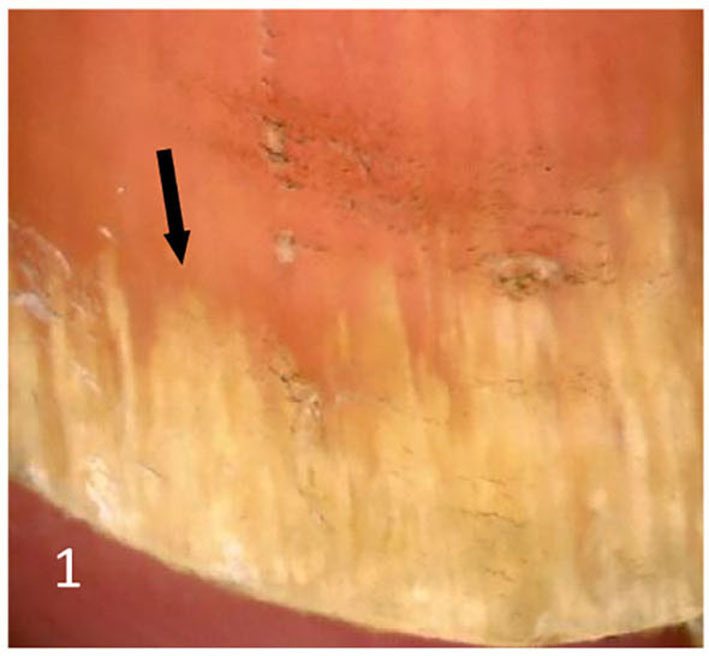
Jagged distal nail plate changes.

**Figure 2:**
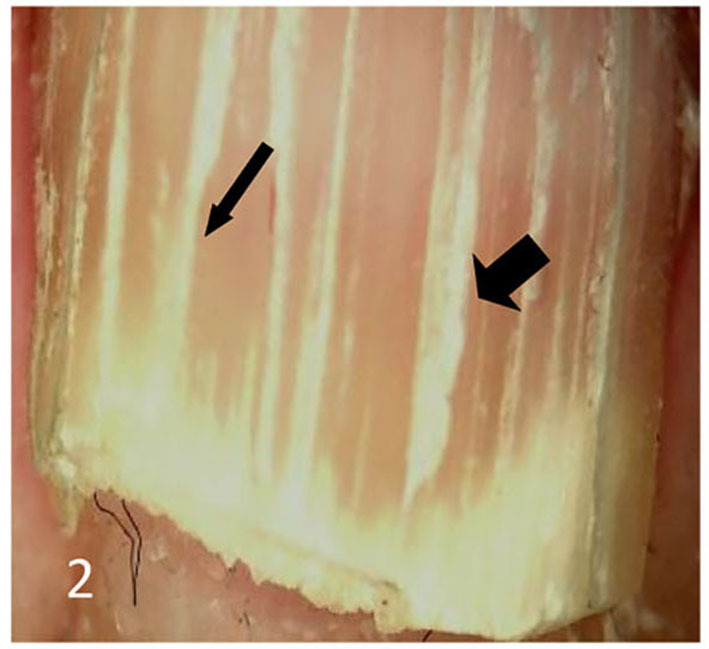
Thin arrows indicate longitudinal striae; thick arrows indicate longitudinal nail fissures.

**Figure 3:**
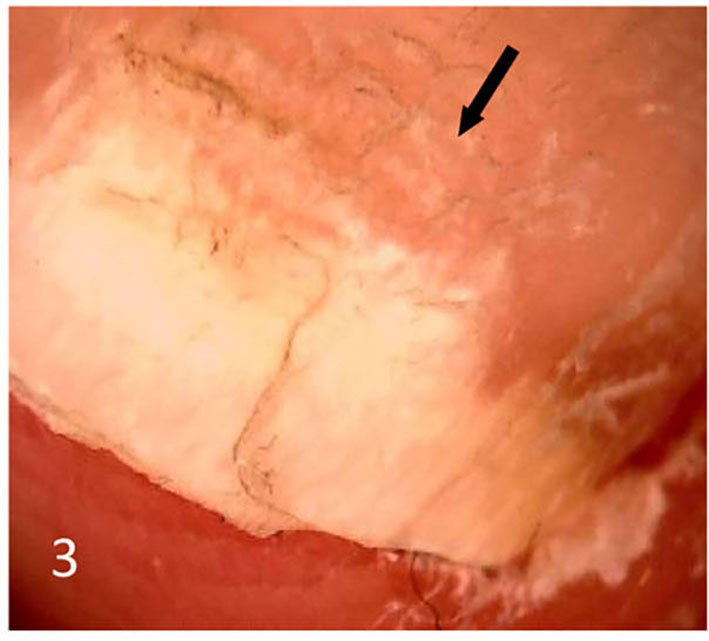
White flocculent nail plate changes.

**Figure 4:**
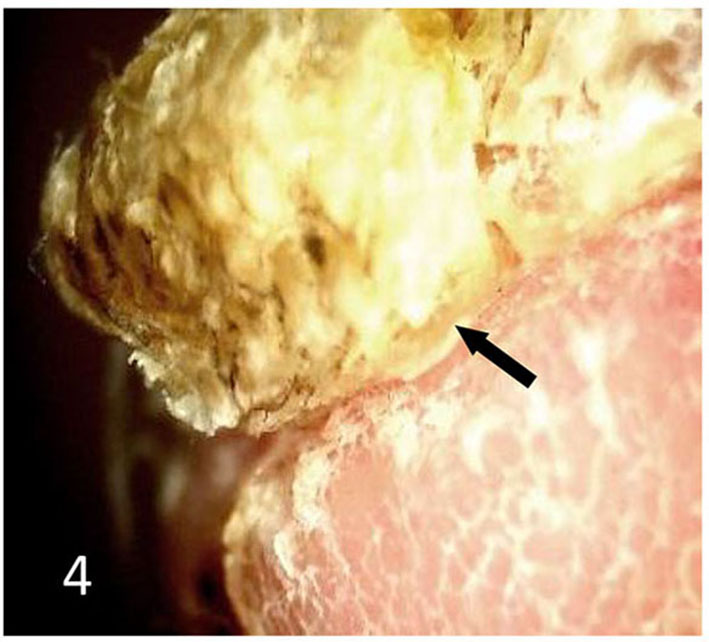
Subungualhyperkeratosis and debris.

**Figure 5:**
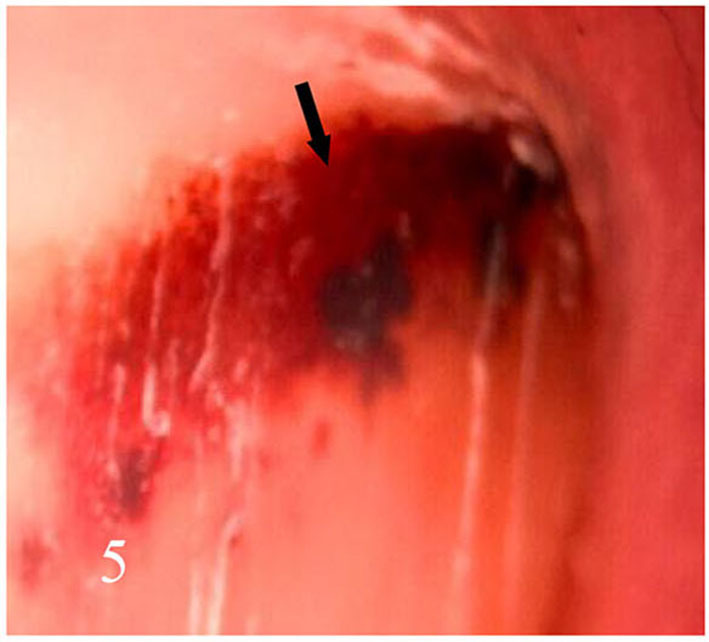
Bleeding under the nail.

**Figure 6:**
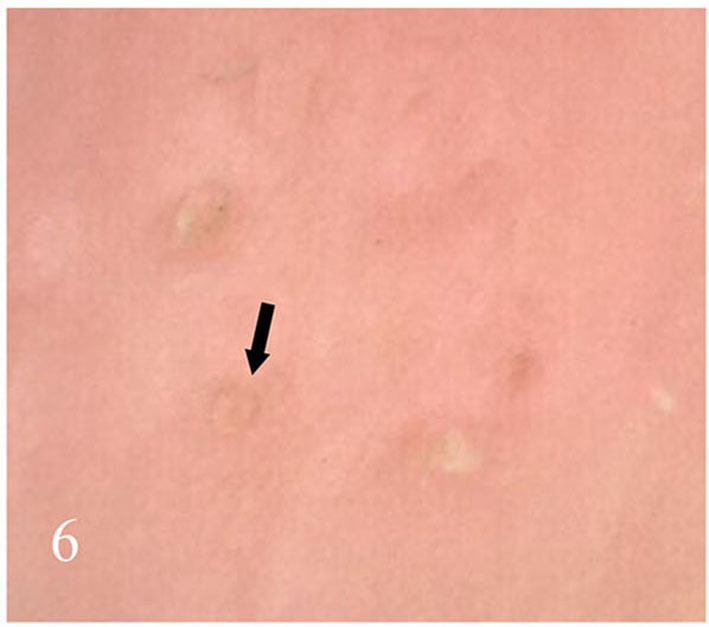
Punctate depression of the nail plate.

**Figure 7:**
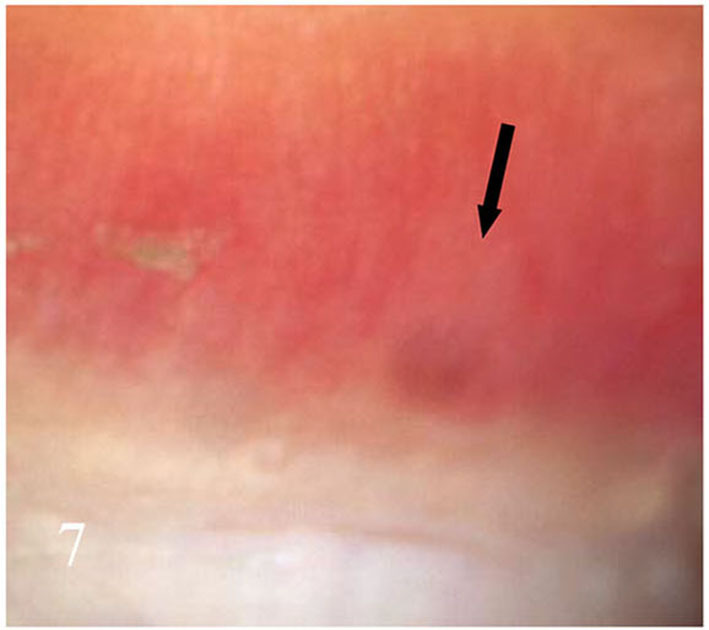
Peri nail erythema.

Dermatoscopic examination and diagnostic basis Macrophotography of the diseased nail and acquisition of dermatoscopic images (×20 non-infiltrating mode) were performed by a dermatoscopic image processing workstation (manufacturer: Guangzhou Chuang Hong Medical Technology Co., Ltd., China, model: CH-DSIS-2000). Selection criteria for dermatoscopic images of onychomycosis: to avoid subjectivity, all dermatoscopic images were analyzed by two physicians and those with consistent results were selected. The diagnosis of onychomycosis was based on two or more of the currently accepted four patterns of dermoscopic examination of onychomycosis: “short spiny pattern, longitudinal striae, distal irregular interrupted pattern and linear pattern” (14). However, in practice, the dermoscopic changes of onychomycosiss were not limited to the above four types. Therefore, the group referred to the dermoscopic patterns of common nail diseases described by Piraccini et al. (14) and Nakamura and Costa (15) and combined with clinical experience, the following nine patterns were selected for observation of the selected diseased nails: serrated distal nail plate changes (Figure 1), longitudinal nail striae (Figure 2), white flocculent changes (Figure 3), subungualhyperkeratosis and debris (Figure 4), longitudinal nail fissures (Figure 2), bleeding under the nail (Figure 5), yellow homogeneous stains, punctate depression deformation (Figure 6), and peri nail erythema (Figure 7).

Inclusion criteria: (1) patients who had not received antifungal medication in the last 6 months; (2) patients with nail changes and positive mycological microscopy (i.e., clear inky filaments observed under the microscope). Exclusion criteria: (1) Patients with co-morbidities such as psoriasis or chronic nail infection that may lead to nail changes; (2) Patients who were unable to cooperate with the retention of clinical and dermoscopic images.

Fungal examination of nail specimens: disinfected the diseased nail with 75% ethanol and scrape the suspected lesion into two equal parts, one for fungal microscopy and one for fungal culture. The collected nail debris specimens were placed on slides with 1 drop of fungal fluorescence staining sdution (Jiangsu Lifetime Biological Technology Co., Ltd., China) mixed thoroughly and left to stand for about 1–3 min after staining and then the slides were covered and observed under a fluorescent microscope (Model: Olympus CX23, Japan, wavelength 340–380 nm) and observed under a fluorescent microscope, positive microscopy was diagnosed by the emission of bright blue fluorescent mycelial structures. This was done by two technicians simultaneously and those with consistent results were included in this study. Inoculate nail scraps in Sabouraud's Medium containing chloramphenicol and actinomycin and incubate at 25°C. Observed and recorded the growth of colonies every other day from the 3rd day after inoculation for a period of 3 weeks.

Statistical methods: The sensitivity and specificity of dermatoscopy and mycological examination were compared with reference to fungal microscopy and culture results. The relationship between dermatoscopic patterns and clinical subtypes was further analyzed in nails diagnosed with onychomycosis. Data were expressed as number of cases and percentages, and all data were expressed as count data, except for the age of the subjects, which was described as mean ± standard deviation. *P* < 0.05 indicated that the difference is statistically significant.

## Results

### Dermatoscopic and Fungal Findings

A total of 114 patients, 68 women and 46 men, aged 40.24 ± 16.33 years old (6–78 years old), with a clinical diagnosis of nail fungal disease were enrolled based on dermoscopic and fungal examination findings. The positive rates of dermoscopy, CFW fungal microscopy and fungal culture in all cases were: 81.58% (93/114), 71.93% (82/114), and 46.49% (53/114), respectively (see Table 1).

**Table 1:** Clinical staging of 114 patients and positivity rate of the three tests.

	**SWO**		**DLSO**		**PSO**		**TDO**		**Total**	
	**#**	**%**	**#**	**%**	**#**	**%**	**#**	**%**	**#**	**%**
Total number of cases	3	2.63	78	68.42	12	10.53	21	18.42	114	
CFW positive	3	2.63	56	49.12	6	5.26	17	14.91	82	71.93
Dermoscopy positive	2	1.75	68	59.65	7	6.14	16	14.04	93	81.58
Culture positive	1	0.88	37	32.45	3	2.63	12	10.53	53	46.49

*Among the 114 patients, the positive rates of dermoscopy, CFW microscopy and fungal culture were 81.58, 71.93, and 46.49% respectively*.

*# Is the number of cases and % is the percentage of the subtype in the total number of lines*.

*SWO, white superficial onychomycosis; DLSO, distal lateral subungual onychomycosis; PSO, proximal subungual onychomycosis; TDO, total dystrophic onychomycosis*.

### Observations of Dermoscopic Sensitivity and Specificity

A positive fungal microscopy and/or culture result of either was used as a positive fungal mycological result to confirm the diagnosis of onychomycosis. This result is also used as a reference to observe the sensitivity and specificity of dermoscopy (Tables 2–4).

**Table 2:** CFW microscopy and fungal culture results.

	**CFW microscopy**	**Fungal cultures**	**Number of cases**
Positive for both	+	+	48
Positive CFW microscopy only	+	–	34
Positive fungal culture only	–	+	5
Negative for both	–	–	27

Based on Table 2, it is clear that there were 87 cases of onychomycosis, the positive rate of fungal microscopy was 82/87 = 94.25% and the positive rate of fungal culture was 53/87 = 60.92%. Among the 114 patients, 87 had positive mycological results and 27 had negative mycological results.

According to Table 3, it can be seen that there were 75 cases with consistent positive dermoscopic and fungal results and 9 cases with consistent negative results, a total of 84 cases, accounting for 96.55% of onychomycosis.

**Table 3:** Dermatoscopic and fungal mycological results.

	**Dermatoscopic**	**Fungal mycological**	**Number of case**
Positive for both	+	+	75
Positive dermoscopy only	+	–	18
Positive fungal examination only	–	+	12
Negative for both	–	–	9

According to Table 4, the sensitivity of dermoscopy for onychomycosis was 86.21%, i.e., dermoscopy easily detects onychomycosis but is not very specific and may misdiagnose nails with similar clinical presentation but caused by other causes as onychomycosis. The positive predictive value (PPV), i.e., the ability of dermoscopy to predict onychomycosis, was 80.65%, which is good. However, the kappa values indicated that the agreement between the two methods was not high, i.e., overall, dermoscopy as a diagnostic method for onychomycosis does not appear to be better than microscopy and culture at present.

**Table 4:** Analysis of the value of dermoscopic testing.

	**Dermoscope (%)**
Sensitivity	86.21
Specificity	33.33
Positive predictive value	80.65
Negative predictive value	42.86
Compliance rate	73.68
Kappa value	21.16

### Distribution of Different Dermoscopic Patterns in Clinical Subtypes of Onychomycosis

As seen in Table 5, of the 87 cases with positive CFW microscopy and positive culture confirming the diagnosis of onychomycosis, the number of cases of each clinical subtype was 3 (3.45%) for white superficial onychomycosis (SWO), 59 (67.82%) for distal lateral subungual onychomycosis (DLSO), 6 (6.90%) for proximal subungual onychomycosis (PSO) and 19 (21.83%) for total dystrophic onychomycosis (TDO), with DLSO and TDO being the most common subtypes, which is consistent with previous literature (5).

**Table 5:** Clinical typing and dermoscopic pattern distribution of 87 patients with onychomycosis.

	**SWO**		**DLSO**		**PSO**		**TDO**		**TOTAL**	
	**#**	**%**	**#**	**%**	**#**	**%**	**#**	**%**	**#**	**%**
Total	3	3.45	59	67.82	6	6.90	19	21.83	87	100
Distal serration	2	2.30	51	58.62	2	2.3	14	16.09	69	79.31
Longitudinal nail stripe	1	1.15	52	59.77	3	3.45	16	18.39	72	82.76
White flocculation	3	3.45	49	56.32	6	6.90	18	20.69	76	87.35
Sub nail keratin build up	0	0	28	32.18	0	0	13	14.94	41	47.13
Longitudinal nail fissures	0	0	11	12.64	0	0	5	5.75	16	18.39
Bleeding under the nail	0	0	17	19.54	0	0	8	9.19	25	28.74
Yellow stain	0	0	34	39.08	1	1.15	11	12.64	46	52.87
Nail plate depression and deformation	1	1.15	21	24.14	0	0	7	8.05	29	33.33
Erythema around the nail	0	0	0	0	1	1.15	3	3.45	4	4.60

*# Is the number of cases, % is the percentage of cases in this group out of 87 cases of nail fungal disease*.

*SWO, white superficial onychomycosis; DLSO, distal lateral subungual onychomycosis; PSO, proximal subungual onychomycosis; TDO, total dystrophic onychomycosis*.

### Intergroup Comparison of Dermoscopic Patterns Between the Two Subtypes of DLSO and TDO

As seen in Table 5, of the four common types of onychomycosis, SWO (3 cases) and PSO (6 cases) were not statistically significant for analysis due to the small number of cases. In contrast, the two clinical subtypes of DLSO (59 cases) and TDO (19 cases) totaled 78 cases, accounting for 89.66% of clinically diagnosed onychomycosis. Therefore, we compared the relationship between dermatoscopic patterns and clinical typing for both DLSO and TDO subtypes and found that *P* > 0.05 (*P* = 0.239), i.e., there was no statistical difference in the distribution of dermatoscopic patterns in these two clinical subtypes (Table 6). However, further analysis of the relationship between the dermatoscopic pattern and clinical staging revealed that nail white flocculation, longitudinal nail striae, jagged changes in the distal nail plate, yellow nail plate staining, and subcutaneous nail keratin accumulation were all more prevalent in the DLSO subtype and the TDO subtype and were the most common dermatoscopic patterns in the clinical setting.

**Table 6:** Distribution of dermoscopic dermatoscopic patterns between two subtypes of DLSO and TDO in 78 cases of onychomycosis.

**ITEM**	**DLSO** **(*n* = 59)**	**TDO** **(*n* = 19)**	***P-*value**
Distal serration	51	14	0.239
Longitudinal nail stripe	52	16	
White flocculation	49	18	
Sub nail keratin build up	28	13	
Longitudinal nail fissures	11	5	
Bleeding under the nail	17	8	
Yellow stain	34	11	
Nail plate depression and deformation	21	7	
Erythema around the nail	0	3	

## Discussion

The incidence of onychomycosis is increasing year on year, accounting for 90% of toenail infections and at least 50% of fingernail infections, and the incidence of onychomycosis varies slightly between age groups. The incidence is 10% in the general population, 20% in those aged 60 years and older, and over 50% in those aged 70 years and older (16–18). Onychomycosis mainly manifests as changes in the color and shape of the nail plate, similar to psoriasis. Nail changes due to inflammatory diseases such as lichen planus, pemphigus, viral warts, and chronic nail fungus are similar, and if the diagnosis is inaccurate it is easy to cause misdiagnosis, thus seriously affecting the quality of life of patients, so a clear diagnosis is very important (19). Direct microscopy with 10% potassium hydroxide (KOH), fungal culture, histopathological assessment, CFW examination, enzyme analysis and polymerase chain reaction are all used in the diagnosis of onychomycosis, with fungal microscopy and culture being the most common and widespread methods, but the sensitivity and specificity of these two methods vary considerably between centers due to the varying levels of testers (20). CFW has a strong affinity for chitin chitinous substance and cellulose of the fungal cell wall and fluoresces bright blue under ultraviolet light for easy identification, so its sensitivity and specificity are significantly higher than KOH microscopy whether it is used for dander, body fluids or nail specimens (21), and has been widely used in fungal microscopy for onychomycosis in China in recent years. Dermatoscopy, as a non-invasive testing tool, can identify structures that are not visible to the naked eye or not easily identified clearly, and is widely used for the diagnosis and differential diagnosis of pigmented skin diseases and certain skin tumors.

It has been confirmed by domestic and international studies (12, 15) that dermatoscopy has been shown to be useful in the diagnosis of onychomycosis, especially in the distal lateral subxiphoid nail type where the dermatoscopic presentation is specific. For patients who are not suitable for fungal microscopy or culture, dermatoscopy is increasingly used to assist in the diagnosis of onychomycosis.

In the 114 patients with suspected onychomycosis in our study, the rate of positive CFW microscopy (71.93%) was higher than that of fungal culture (46.49%), in agreement with that reported by Dass et al. (22), and the rate of positive dermoscopy (81.58%) was also slightly higher than that of CFW microscopy. When fungal mycological results were used as a reference, the sensitivity of dermatoscopy was 86.21%, but the specificity was 33.33%, which may be influenced by the combined drawbacks of the respective dermatoscopic and pathogenic examinations. The tendency for false positives in dermatoscopic diagnosis due to the similar clinical presentation of nail damage from various causes and the limitations of nail specimens taken during pathogenic examination are the main reasons for their false negatives. The high positive predictive value and low negative predictive value of dermoscopy further suggest that dermoscopy is comparable to CFW microscopy in terms of sensitivity, although less specificity. However, as a non-invasive diagnostic imaging technique, it is still a good option for patients for whom nail specimen sampling is inconvenient and for primary screening and condition monitoring of onychomycosis.

The dermatoscopic pattern of onychomycosis and its differentiation from other nail diseases has been reported (23, 24), but relatively few studies have addressed the presentation of the dermatoscopic pattern in different clinical subtypes. Piraccini et al. (14) first proposed that the characteristic dermoscopic pattern of DLSO onychomycosis is a serrated edge at the junction of the diseased nail and the normal nail with the tip facing the nail bed, while the characteristic pattern of traumatic nail injury is a wavy edge at the border of the diseased nail and the normal nail. However, the dermatoscopic patterns of the other three clinical subtypes of onychomycosis are still inconclusive. Jesus-Silva et al. (25) analyzed the correlation between KOH microscopy and dermoscopic patterns in 178 diseased nails with clinically suspected onychomycosis and found that longitudinal nail stripes and serrated edges were more commonly associated with DLSO or TDO subtypes, and the incidence of positive distal serrated patterns in nails with pathogenically confirmed DLSO subtypes in this study was only 43.59%, which is inconsistent with Piraccini's et al. (14) who concluded that the sensitivity of the serrated pattern in the DLSO subtype was 100%. The present study also found a high rate of positivity for the distal serrated margin and nail longitudinal pattern in the DLSO subtype and TDO subtype, in agreement with the Jesus-Silva findings (25). However, the statistical analysis of the longitudinal nail striae and the distal serrated edge of the nail plate showed no statistically significant difference between the DLSO and TDO subtypes (*P* > 0.05), in agreement with Jesus-Silva's conclusions (25) but not with Chetana et al. (26), which may be related to the small number of cases included. Since DLSO is most common clinically and can eventually progress to TDO, distal serrated margins and longitudinal nail striae are also highly positive in the TDO subtype (14/19, 16/19). The yellow stain pattern is characterized by a stained yellow homogeneous change in the nail plate, while the subcutaneous keratin accumulation pattern refers to a ruinous accumulation of keratin on the ventral side of the nail plate (27). In the present study these two patterns were also more positive in the DLSO and TDO subtypes, and the statistical difference in dermoscopic pattern between these two subtypes was not statistically significant, which still needs to be confirmed in a large sample study. In addition, white flocculent changes of the nail plate are usually a clinical feature of the SWO type, and we found that this pattern, in addition to being seen in all SWO cases (3/3), also had a high positive rate in the DLSO subtype (49/59) and TDO subtype (18/19), with this dermoscopic pattern accounting for 87.35% of all onychomycosiss, suggesting that the latter two types of onychomycosis are highly susceptible to coexistence with the SWO subtype. We also found a case of PSO subtype with a white nail involving the proximal 4/5 of the nail plate and a serrated edge with the tip toward the free edge of the nail on the lateral edge of the white nail, as reported by Yorulmaz and Yalcin (27). In summary, we suggest that the longitudinal stripe pattern, the distal serrated pattern and the sub nail cuticle accumulation pattern are more likely to occur in the DLSO and TDO subtypes, as in the existing reports (25–27), whereas the yellow stain pattern is mostly used for the microscopic description of onychomycosis in the existing reports, and its diagnostic value is debatable.

This paper is the pioneer in comparing the value of dermoscopy and mycological examination in the diagnosis of onychomycosis, which is less well reported in the existing literature. In addition, the occurrence of nine dermoscopic patterns in different clinical subtypes of onychomycosis was analyzed, and it was concluded that dermoscopy can be a rapid, non-invasive and effective tool for the diagnosis of onychomycosis by combined fungal pathogenesis. The correlation between dermatoscopic patterns and clinical subtypes of onychomycosis needs to be further investigated in large samples.

## Data Availability Statement

The original contributions presented in the study are included in the article/supplementary material, further inquiries can be directed to the corresponding authors.

## Ethics Statement

The studies involving human participants were reviewed and approved by the Local Medical Ethics Committee. The patients/participants provided their written informed consent to participate in this study. Written informed consent was obtained from the individual(s) for the publication of any potentially identifiable images or data included in this article.

## Author Contributions

YM and WF gave sufficient guidance to this study. All authors of this study have made equal contributions, including study design, inclusion of cases, implementation of experiments, statistics of data, and writing of the paper. All authors contributed to the article and approved the submitted version.

## Funding

This work was supported by the Youth Science and Technology Research Fund of Shanxi Province Applied Basic Research Program (201901D211499) and the New Technology New Project of the Second Hospital of Shanxi Medical University (2017021).

## Conflict of Interest

The authors declare that the research was conducted in the absence of any commercial or financial relationships that could be construed as a potential conflict of interest.

## Publisher's Note

All claims expressed in this article are solely those of the authors and do not necessarily represent those of their affiliated organizations, or those of the publisher, the editors and the reviewers. Any product that may be evaluated in this article, or claim that may be made by its manufacturer, is not guaranteed or endorsed by the publisher.
